# Yersiniabactin produced by *Escherichia coli* promotes intestinal inflammation through lipid peroxidation and ferroptosis

**DOI:** 10.3389/fmicb.2025.1542801

**Published:** 2025-02-17

**Authors:** Hao Wang, Bingxun Chen, Peng Xiao, Dongmei Han, Bin Gao, Yulin Yan, Ru Zhao, Tianling Pan, Jingsong Zhang, Meng Zhou, Longbao Lv, Hong Gao

**Affiliations:** ^1^College of Food Science and Technology, Yunnan Agricultural University, Kunming, China; ^2^College of Veterinary Medicine, Yunnan Agricultural University, Kunming, China; ^3^National Resource Center for Non-Human Primates, National Research Facility for Phenotypic and Genetic Analysis of Model Animals (Primate Facility), Kunming Institute of Zoology, Chinese Academy of Sciences, Kunming, China

**Keywords:** *Escherichia coli*, yersiniabactin, lipid peroxidation, ferroptosis, intestinal inflammation

## Abstract

*Escherichia coli* (*E. coli*), a major foodborne pathogen, poses significant risks to public health by causing gastrointestinal diseases. Among its virulence factors, Yersiniabactin (Ybt), a siderophore, plays a crucial role in iron acquisition and enhancing intestinal colonization. Despite previous studies highlighting *E. coli*-Ybt’s involvement in inflammation, its exact mechanisms remain unclear. This study investigates how Ybt contributes to intestinal inflammation through ferroptosis, using both *in vitro* and *in vivo* models. Our findings demonstrate that Ybt promotes oxidative stress, lipid peroxidation, inflammation, and iron accumulation in intestinal epithelial cells, leading to ferroptosis. Mechanistically, Ybt suppresses the Keap1/Nrf2 pathway, amplifying reactive oxygen species (ROS) and activating the TNF/NF-κB pathway, which drives inflammation. Moreover, Ybt induces lipid peroxidation via the arachidonic acid pathway, producing 6-trans-leukotriene B4 (6-transLTB4), which exacerbates inflammation and ferroptosis. Exogenous 6-transLTB4 further intensifies this cascade. Additionally, Ybt disrupts iron efflux by suppressing FPN1 expression, causing excessive intracellular iron accumulation. Using tree shrews as an *in vivo* model, we confirm that Ybt-induced ferroptosis significantly aggravates intestinal inflammation. These findings underscore the pathogenic role of Ybt in *E. coli*-induced intestinal injury and highlight ferroptosis as a novel mechanism contributing to gut health disruption. This study provides new insights into the molecular pathways of *E. coli* infection, with implications for therapeutic strategies targeting ferroptosis in intestinal diseases.

## 1 Introduction

*Escherichia coli* (*E. coli*), a significant zoonotic pathogen, is a leading cause of foodborne illnesses (Wang et al., 2024). It adheres to the intestine through pili proteins and subsequently causes various gastrointestinal diseases in humans, such as diarrhea and hemorrhagic colitis (Govindarajan et al., 2020; Govindarajan and Kandaswamy, 2022; Wang et al., 2024). Infections often result from consuming contaminated food or water, underscoring the importance of understanding *E. coli*’s effects on gut health. While most *E. coli* strains are harmless, some acquire the high pathogenicity island (HPI) via horizontal gene transfer, enabling the production of Yersiniabactin (Ybt) (Koh et al., 2017). This siderophore enhances *E. coli*’s intestinal colonization and virulence (Sobieszczańska, 2008), contributing to severe gastrointestinal infections that can escalate to systemic organ failure and fatal outcomes (Bachman et al., 2011; Garcia et al., 2011; Ellermann et al., 2019). Previous studies have focused on Ybt-induced inflammatory responses, including elevated levels of tumor necrosis factor-α (TNF-α) and interleukin-1β (IL-1β) (Liu et al., 2018). Ybt has also been shown to induce autophagy and activate the NLRP3 inflammasome, leading to pyroptosis (Dalmasso et al., 2021; Wang et al., 2023b).

Ferroptosis is a distinct form of cellular demise, distinguishing itself from other modalities such as autophagy and pyroptosis (Dixon et al., 2012; Jiang et al., 2021; Zhang et al., 2023). It is characterized by intracellular phospholipid peroxidation and iron accumulation, influenced by phospholipids containing polyunsaturated fatty acid chains (PUFA-PL) and transition metals. Various signals within and between cells, along with environmental factors affecting cell metabolism and levels of reactive oxygen species (ROS), regulate ferroptosis (Bersuker et al., 2019; Jiang et al., 2021). The regulatory pathways governing ferroptosis include the glutathione/glutathione peroxidase 4 (GSH/GPX4) pathway (Ingold et al., 2018; Doll et al., 2019), the lipid metabolism pathway (Rochette et al., 2022), and the iron metabolism regulation pathway associated with ferroptosis (Stockwell et al., 2017; Stockwell, 2022). Notably, *E. coli* infections are known to trigger oxidative stress and lipid peroxidation (Portolés et al., 1993; Fock et al., 2018), suggesting that ferroptosis may represent a novel mechanism by which Ybt contributes to intestinal injury.

This study explores the link between Ybt and intestinal inflammation using tree shrews as an *in vivo* model. Our findings highlight the critical role of Ybt-induced ferroptosis in driving oxidative stress, lipid peroxidation, and iron accumulation, contributing to intestinal damage. These results further highlight the potential risk of pathogenic *E. coli* to intestinal health and may provide additional valuable evidence for understanding *E. coli* damage to human gut.

## 2 Materials and methods

### 2.1 Cell culture

FHs 74 Int cells, derived from human small intestinal epithelial cells (Guangzhou Otwo Biotech, Guangzhou, China), were used to model *in vitro* infection. These cells were cultured in a medium supplemented with 10% FBS, penicillin, and streptomycin. Subsequently, cells were maintained in an incubator at 37°C with 5% CO_2_.

### 2.2 Bacterial strains

*Escherichia coli*-Tb is a clinically pathogenic strain isolated from the feces of a tree shrew with diarrhea in Yunnan, China, is classified as enteropathogenic *E. coli* (EPEC). This strain harbors the HPI and produces Ybt.

### 2.3 Construction of *Escherichia coli*-*irp2* gene knockout and complement strains

In this study, TbΔ*irp2* was generated using CRISPR/Cas9 gene editing methodology, following a pre-established protocol (Jiang et al., 2015; Wang et al., 2023a). TbΔ*irp2*(Ybt^−^) is a mutant strain derived from *E. coli*-Tb (Ybt^+^), with the deletion of the *irp2* gene leading to the loss of Ybt production.

The full open reading frame (ORF) of the *irp2* gene was cloned into the pBAD33 plasmid. This recombinant plasmid was introduced into the *irp2*-deleted strain to create a complemented strain, C-TbΔ*irp2*. All strains (*E. coli*-Tb, TbΔ*irp2*, and C-TbΔ*irp2*) were cultured in LB medium at 37°C.

### 2.4 Animal experiments

Thirty male tree shrews (190 ± 10 g, approximately 1.5 years old) were obtained from the Kunming Institute of Zoology, Chinese Academy of Sciences. The animals were housed in a temperature-controlled facility under a 12-h light/dark cycle with ad libitum access to food.

To induce intestinal infection, tree shrews were intraperitoneally (i.p.) injected with a bacterial solution at a concentration of 1 × 10^7^ CFU/animal. The animals were randomly assigned to four groups (*n* = 3 per group): Control (PBS only), *E. coli*-Tb, TbΔ*irp2*, and C-TbΔ*irp2*.

For metabolite pro-inflammatory studies, 6-transLTB4 (Santa Cruz Biotechnology, Inc., #71652-82-9) was administered intraperitoneally at a dose of 0.5 mg/kg, 3 h after *E. coli* infection. The 6-transLTB4 stock solution in DMSO was diluted with PBS to achieve a final concentration of 0.5 mg/mL. The tree shrews were divided into six groups (*n* = 3 per group): Control group (PBS only), 6-transLTB4 group (6-transLTB4 injection only), *E. coli*-Tb group (*E. coli*-Tb infection only), *E. coli*-Tb^+^ 6-transLTB4 group (*E. coli*-Tb infection followed by 6-transLTB4 injection), TbΔ*irp2* group (TbΔ*irp2* infection only), TbΔ*irp2*^+^ 6-transLTB4 group (TbΔ*irp2* infection followed by 6-transLTB4 injection). Animals were euthanized 24 h post-treatment, and intestinal samples were collected for further analysis.

After the experimental animals were treated in different ways, we collected intestinal samples after euthanizing the animals 24 h later. This study received ethical approval from the Animal Ethics Committee of Yunnan Agriculture University (202303006) and adhered to the ARRIVE guidelines.

### 2.5 Cells experiments

For the infection experiment, 2 × 10^6^ FHs 74 Int cells were seeded into culture plate wells, followed by the addition of *E. coli* at a multiplicity-of-infection (MOI) of 20:1. The experiment comprised four groups: Control, *E. coli*-Tb, TbΔ*irp2*, and C-TbΔ*irp2*. The Control group received sterile PBS only, while the other groups were treated with the corresponding *E. coli* strains at the predetermined dose.

For the metabolite validation experiment, 10 nM 6-transLTB4 was added to the cultured cells. Six experimental groups were established, mirroring the *in vivo* experimental setup: Control (PBS), 6-transLTB4 only, *E. coli*-Tb, *E. coli*-Tb^+^ 6-transLTB4, TbΔ*irp2*, and TbΔ*irp2*^+^ 6-transLTB4. 6-transLTB4 was administered 0.5 h after infection with the respective *E. coli* strains at the predetermined dose.

Cells and cell supernatants were collected at designated time points (8 h post-infection) for subsequent analysis. All cell experiments were performed with a minimum of three biological replicates.

### 2.6 Enzyme-linked immunosorbent assay

The concentrations of TNF-α (Mlbio, # ml077385) and IL-1β (Mlbio, # ml058059) in cell samples were determined utilizing an enzyme-linked immunosorbent assay (ELISA) kit. All procedures were carried out following the manufacturer’s protocols.

### 2.7 Lipid peroxidation assay

Cellular lipid peroxidation levels were assessed using specific lipid peroxidation detection kits, namely LPO (Jiancheng Bioengineering, Nanjing, China), MAD (Boxbio Co., Ltd.), SOD (Boxbio Co., Ltd.), CAT (Boxbio Co., Ltd.), and GSH-Px (Jiancheng Bioengineering, Nanjing, China). All procedures were conducted following the manufacturer’s protocols. Each experiment was replicated three times.

### 2.8 ROS assay

To quantify ROS levels in cell samples, a ROS-ELISA kit (#AKCE002-2, Beijing Boxbio Science & Technology, Nanjing, China) was used according to the manufacturer’s instructions.

For fluorescence imaging of intracellular ROS, the ROS Red fluorescence detection kit (#CA1420, Solarbio) was employed. Cells were treated with DHE red fluorescent dye (15 μM) and incubated at 37°C in the dark for 30 min. After incubation, the staining solution was removed, and the cells were washed twice with PBS. ROS levels were then visualized using fluorescence microscopy.

### 2.9 Assessment of iron levels

Iron concentrations in cell lysates and intestine were determined utilizing an iron detection kit (Sigma-Aldrich, #025), adhering to the manufacturer’s instructions.

### 2.10 Ferroptosis assay

FHs 74 Int cells were treated with *E. coli* for 12 h, with or without ferrostatin-1 (Fer-1, 0.4 μM). Additionally, to validate oxidative lipid metabolites, FHs 74 Int cells were exposed to *E. coli* and 6-transLTB4 alone or in combination for 12 h, with or without ferrostatin-1 (Fer-1, 0.4 μM). Cell death was quantified by measuring LDH release utilizing the CytoTox-ONE^™^ Cytotoxicity Detection Kit (Promega, #G7890).

### 2.11 Immunohistochemical assay

Duodeno-intestinal specimens were fixed in a 4% paraformaldehyde solution for 24 h, embedded in paraffin, and sectioned into 5 μm slices using the Leica RM2125 RTS slicing mechanism. Immunohistochemical assay (IHC) analysis included repairing the intestinal tract sections with eBioscience^™^ IHC (Thermo Fisher, # 00-4956-58) antigen retrieval solution, followed by a 1-h incubation with 5% BSA to block nonspecific antigens. Specific antibodies against IL-6 (#66146-1-Ig, Proteintech), IL-18 (#10663-1-AP, Proteintech), IL-1β (#16806-1-AP, Proteintech), TNF-α (#60291-1-Ig, Proteintech), and SLC40A1 (#26601-1-AP, Proteintech) were then used for staining. Subsequently, incubation with HRP-labeled secondary antibodies was followed by DAB color development.

### 2.12 qPCR analysis

RNA was extracted from cells in different treatment groups using RNAiso Plus (Trizol, # 9109); the extracted RNA was reverse transcribed into cDNA following the instructions of the reverse transcription kit. The mRNA levels of the target genes in cells were detected by qPCR, utilizing relative quantification to assess the mRNA levels of each gene within the respective groups. Primer information is provided in Supplementary Table S1.

### 2.13 Western blot

After washing the cells with PBS, proteins were extracted using RIPA lysis buffer. Protein concentrations were determined using a BCA kit. A 30 μg protein sample was separated on a 10% SDS-PAGE gel and transferred onto a PVDF membrane. The membrane was blocked with a protein-free rapid blocking solution and incubated overnight at 4°C with specific primary antibodies: ACSL4 (Proteintech, 22401-1-AP), SLC7A11 (Proteintech, 26864-1-AP and 26601-1-AP), GPX4 (Proteintech, 30388-1-AP). Protein bands were visualized using enhanced chemiluminescence (ECL) staining after incubation with species-specific secondary antibodies. Beta-actin was used as a loading control.

### 2.14 RNA-seq analysis

Cells were collected 8 h post-bacterial infection according to established protocols. RNA was extracted and stored at −80°C until subjected to RNA-Seq analysis using a BGISEQ-500 sequencing platform provided by BGI Company, China. Differentially expressed genes (DEGs) were identified using DEGseq (fold change ≥2 and adjusted *p*-value ≤0.01). Enrichment analyses of the DEGs were performed for Gene Ontology (GO) and Kyoto Encyclopedia of Genes and Genomes (KEGG) using OmicShare tools.^1^ Heatmaps and volcano plots were generated to illustrate DEG expression based on log2 FPKM values.

### 2.15 Targeted oxidative lipidomics

The AB Sciex QTRAP 6500 MetWare LC-ESI-MS/MS platform^2^ was utilized to analyze the content of oxidative metabolites derived from polyunsaturated fatty acids, including ARA, linoleic acid, α-linolenic acid, DHA, EPA, DGLA, among others. Differential metabolites between groups were identified based on VIP and absolute Log2FC. GO and KEGG enrichment analysis of differential metabolites were conducted utilizing the Metware Cloud tools.^3^

### 2.16 Molecular docking

FPN1 (PDB: 6W4S) from the RCSB PDB database was employed as the receptor protein, while Ybt (CID: 443589) from PubChem was utilized as the small molecule ligand. Subsequently, molecular docking was conducted using CB-Dock2 (Liu et al., 2022).

### 2.17 Statistical analysis

Statistical analysis was based on a minimum of three biological replicates. Data were expressed as mean ± SD and analyzed using either Student’s t-test or two-way ANOVA. Significance was defined as *p*-values less than 0.05.

## 3 Results

### 3.1 Ybt promotes intestinal inflammation caused by *Escherichia coli*

Ybt production is directed by HPI, which comprises the functional core region formed by the *irp2*-FyuA gene axis (Carniel et al., 1996) Mutations in the *irp2* gene disrupt Ybt production (Magistro et al., 2017). To investigate Ybt’s role in *E. coli* pathogenesis, we knocked out the *irp2* gene in *E. coli*-Tb. The gene structure of HPI is shown in Supplementary Figure S1A. Using two sgRNAs, we targeted the *irp2* gene for editing, as illustrated in Supplementary Figure S1B. Subsequently, we confirmed the creation of TbΔ*irp2* through PCR and gene sequencing (Supplementary Figure S1C). In addition, we followed the procedure of Supplementary Figure S2 to construct the corresponding mutant complement strain and named it C-TbΔ*irp2*.

**Figure 1:**
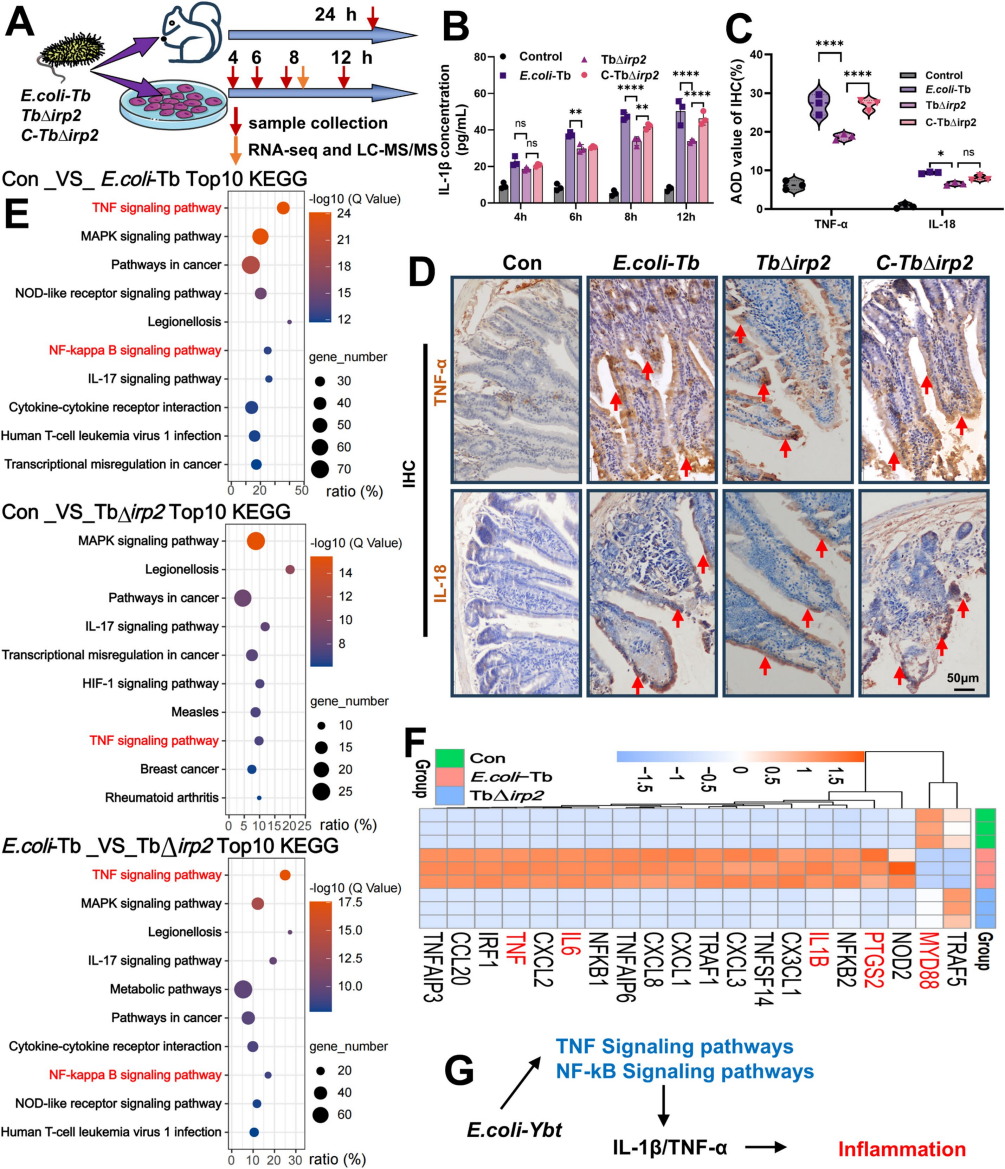
Ybt promotes intestinal inflammation caused by *E. coli*. **(A)** Schematic diagram of the Ybt infection model. **(B)** Changes in IL-1β content in cell supernatant after infection, *n* = 3. **(C,D)** IHC findings of the intestinal tract of tree shrews after different treatments (scale: 50 μm; arrows indicate typical damage) and quantification of the mean optical density of the results, *n* = 3. **(E)** Top 10 KEGG analysis of differentially expressed DEGs between different groups (pathways related to inflammatory response highlighted in red font). **(F)** Heatmap depicting the expression of inflammation-related genes following various treatments. **(G)** Schematic illustration of the inflammatory mechanism caused by Ybt. ^**^p < 0.01 and ^****^p < 0.0001.

We established *E. coli* infection models using enterocytes and tree shrews (Figure 1A). Analysis of inflammatory markers revealed that, starting at 6 h post-infection, *E. coli*-Tb strains capable of secreting Ybt induced significantly higher levels of IL-1β in cells compared to strains lacking Ybt (TbΔ*irp2*). This reduction in IL-1β release in TbΔ*irp2*-infected cells was reversed upon infection with the complement strain C-TbΔ*irp2* (Figure 1B). In addition, we observed the levels of inflammatory factors (TNF-α and IL-18) in the intestinal tract of tree shrews, and similar results were observed in intestinal epithelial cells (Figures 1C,D). These results show that *E. coli* strains capable of secreting Ybt exhibited heightened inflammatory-inducing activity in the intestinal tract.

RNA-seq analysis was performed on intestinal epithelial cells infected with *E. coli*-Tb and TbΔ*irp2* to delve deeper into the mechanism of Ybt in promoting intestinal inflammation. KEGG analysis of the enriched DEGs revealed that the majority of the top 10 pathways following infection were associated with inflammation (Figure 1E). Particularly, *E. coli*-Tb infection resulted in a greater enrichment of inflammatory pathways compared to TbΔ*irp2* infection (Figure 1E). The TNF pathway and NF-kB pathway are typical inflammatory pathways (Hsu et al., 1995; Tang et al., 2009), and our analysis revealed that Ybt contributed to the enrichment of numerous inflammatory DEGs, many of which were key components of the TNF and NF-κB pathways (Figure 1F). These findings suggest that Ybt augments the pro-inflammatory traits of *E. coli* and contributes to *E. coli*-induced intestinal inflammation via the TNF/NF-κB pathway (Figure 1G).

### 3.2 Ybt promotes oxidative stress injury in intestinal epithelial cells

**Figure 2:**
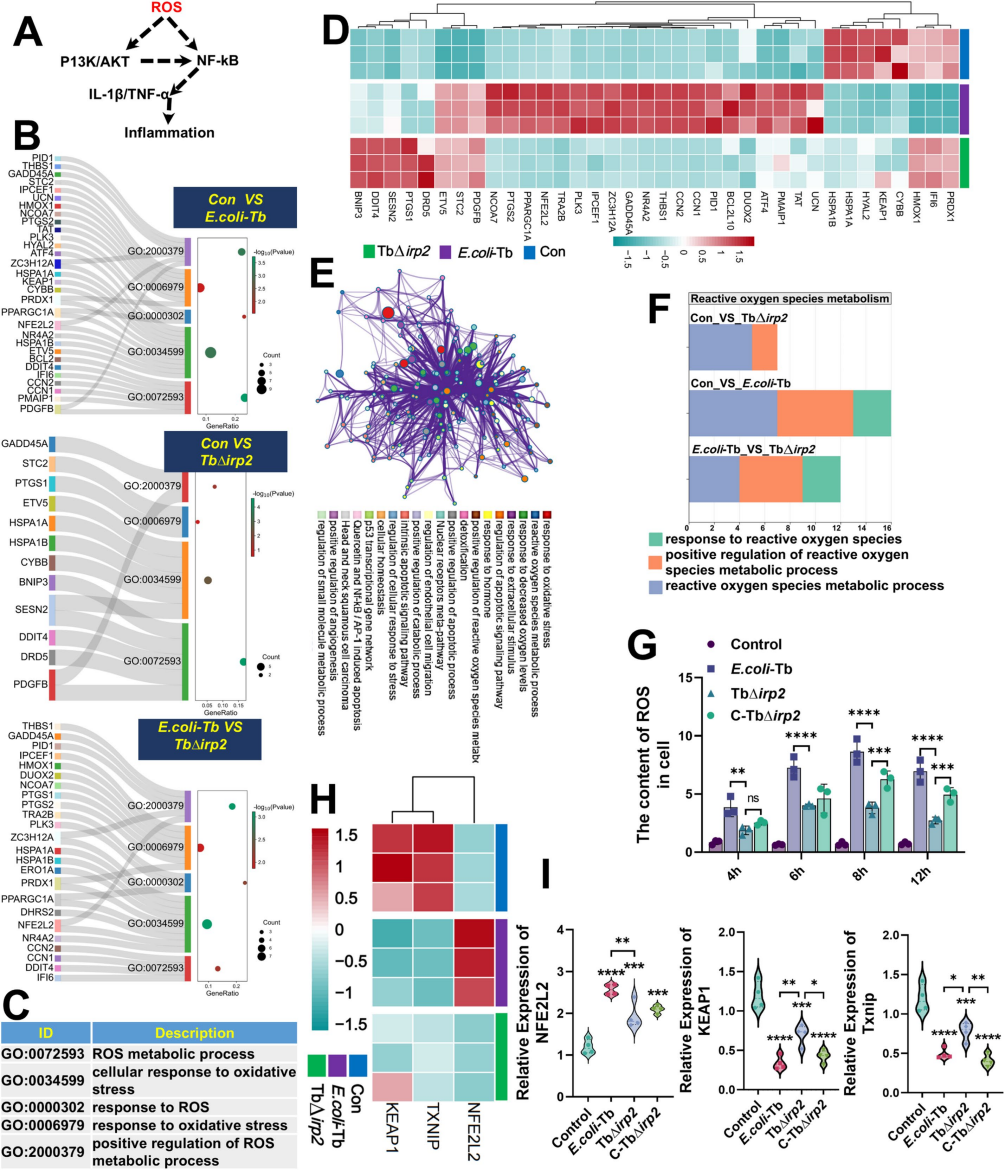
Ybt promotes oxidative stress injury. **(A)** Schematic representation illustrating how ROS promotes inflammatory responses. **(B,C)** GO analysis of DEGs associated with oxidative stress following various treatments. **(D)** Cluster heatmap showing DEGs related to oxidative stress after different treatments. **(E)** The Metascape tool was utilized to analyze the pathway network of DEGs associated with oxidative stress. **(F)** GO enrichment was conducted to explore the correlation between Ybt and ROS generation. **(G)** The levels of ROS in the cells were measured using ELISA, *n* = 3. **(H)** A cluster heatmap was generated to visualize the DEGs related to ROS generation after different treatments. **(I)** qPCR was employed to ascertain the mRNA levels of *NFE2L2*, *KEAP1*, and *Txnip*, *n* = 4. ^*^p < 0.05, ^**^p < 0.01, ^***^p < 0.001, and ^****^p < 0.0001.

ROS serves as a pivotal regulator of the TNF/NF-κB proinflammatory signaling pathway (Pham et al., 2004) (Figure 2A). Considering the exacerbating effect of Ybt on intestinal inflammation, we hypothesized that it might be associated with ROS-mediated oxidative stress. RNA-seq GO analysis revealed the participation of ROS and oxidative stress pathways in *E. coli* infection, with a greater enrichment of DEGs related to oxidative stress in *E. coli*-Tb infection compared to TbΔ*irp2* (Figures 2B,C). Additionally, infection with *E. coli*-Tb markedly increased the up-regulation or down-regulation of oxidative stress-related DEGs compared to TbΔ*irp2* infection (Figure 2D). Further validation and annotation of KEGG functions were performed through functional annotation of DEGs using Metascape, revealing enrichment of the top 20 clusters of biological processes, including “response to oxidative stress” and “reactive oxidative stress metabolic process” (Figure 2E). These findings suggest that *E. coli*-Tb induces oxidative stress injury in intestinal epithelial cells.

ROS-mediated oxidative stress plays a crucial role in the inflammatory process (Pham et al., 2004; Morris et al., 2022). Consequently, GO enrichment analysis of ROS-associated pathways following *E. coli* infection was conducted, indicating a greater number of ROS-associated DEGs in *E. coli*-Tb infection compared to TbΔ*irp2* infection (Figure 2F). To further investigate this, we measured ROS levels in intestinal epithelial cells. The results revealed a significant increase in ROS levels during *E. coli*-Tb infection. In contrast, TbΔ*irp2* infection reduced ROS overproduction. However, infection with the complement strain C-TbΔ*irp2* restored ROS levels to those observed during *E. coli*-Tb infection (Figure 2G).

The Keap1/Nrf2-ARE pathway is essential for maintaining homeostasis by regulation of oxidative stress (DeNicola et al., 2011; Hayes and Ashford, 2012). RNA-seq analysis shows that Ybt enhanced the inhibition of this pathway, corroborated by qPCR results (Figures 2H,I). Consequently, the induction of oxidative stress injury in intestinal epithelial cells by Ybt is linked to Ybt’s inhibition of the Keap1/Nrf2 pathway, resulting in elevated ROS levels.

### 3.3 Ybt induces ferroptosis in intestinal epithelial cells

**Figure 3:**
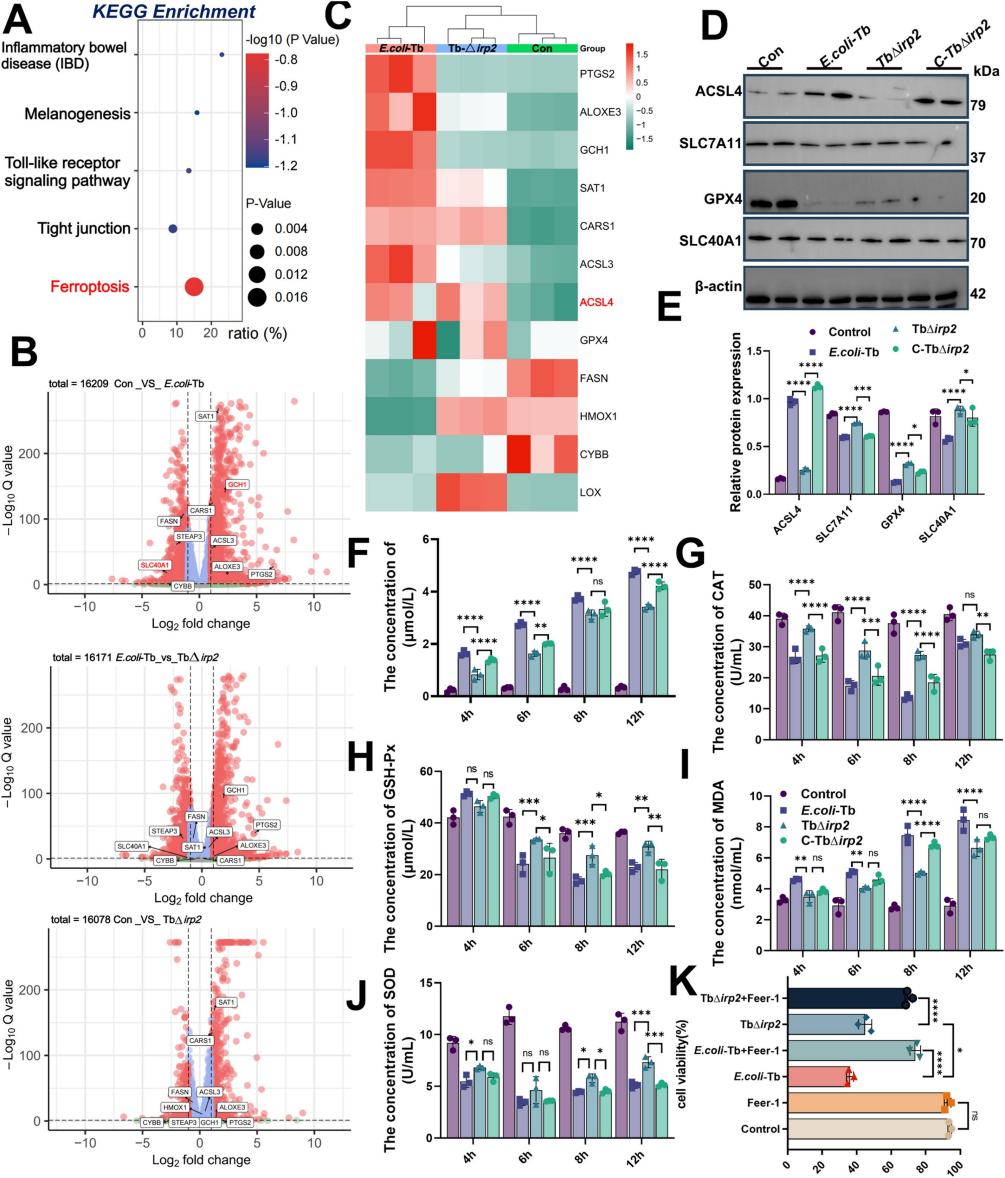
Ybt induces ferroptosis in intestinal epithelial cells. **(A)** KEGG analysis was employed to investigate the association between Ybt and ferroptosis. **(B)** Volcano plots were generated to compare differential fold changes and *p*-values for individual genes after different treatments. **(C)** Clustering heatmaps were created to visualize the DEGs associated with ferroptosis after different treatments. **(D,E)** Western blot was used to detect the expression levels of iron death marker proteins after different treatments, including ACSL4, SLC7A11, GPX4, and SLC7A1, at the same time, the protein expression was quantitatively analyzed. **(F–J)** Intracellular lipid peroxide content was quantified at various time points following diverse treatments, including LPO, CAT, GSH-Px, MDA and SOD, *n* = 3. **(K)** The impact of the ferroptosis inhibitor Fer-1 on cell viability was evaluated following various treatments, *n* = 3. ^*^p < 0.05, ^**^p < 0.01, ^***^p < 0.001, and ^****^p < 0.0001.

Ferroptosis is closely linked to ROS accumulation (Yang et al., 2023). Given the augmentation of oxidative stress by Ybt, we delved deeper into the association between Ybt and ferroptosis. RNA-seq KEGG enrichment analysis indicates the involvement of ferroptosis in *E. coli*-Tb infection (Figure 3A). Subsequent volcano plot and heatmap enrichment analysis of DEGs demonstrate notable upregulation or downregulation of ferroptosis-related genes in *E. coli*-Tb infection compared to TbΔ*irp2* infection, as validated by qPCR (Figures 3B,C; Supplementary Figure S3). Additionally, we assessed the expression levels of ferroptosis marker proteins following *E. coli* infection. Ybt upregulated the expression of acyl-CoA synthetase long-chain family member 4 (ACSL4). Conversely, the expression of solute carrier family members SLC7A11, glutathione peroxidase 4 (GPX4), and the iron efflux pump protein FPN1/SLC40A1 was downregulated, with this effect alleviated in the TbΔ*irp2* infection group. These changes were reversed in the C-TbΔ*irp2* infection group (Figures 3D,E).

Lipid peroxidation significantly contributes to the occurrence and progression of ferroptosis (Nishizawa et al., 2021). Findings demonstrate that *E. coli*-Tb infection elevates lipid peroxidation in cells, whereas this effect is diminished in TbΔ*irp2*-infected cells (Figures 3F–J). Importantly, Fer-1, a ferroptosis inhibitor, alleviated Ybt-induced cell death (Figure 3K). These findings indicate that Ybt effectively trigger ferroptosis in intestinal epithelial cells.

**Figure 4:**
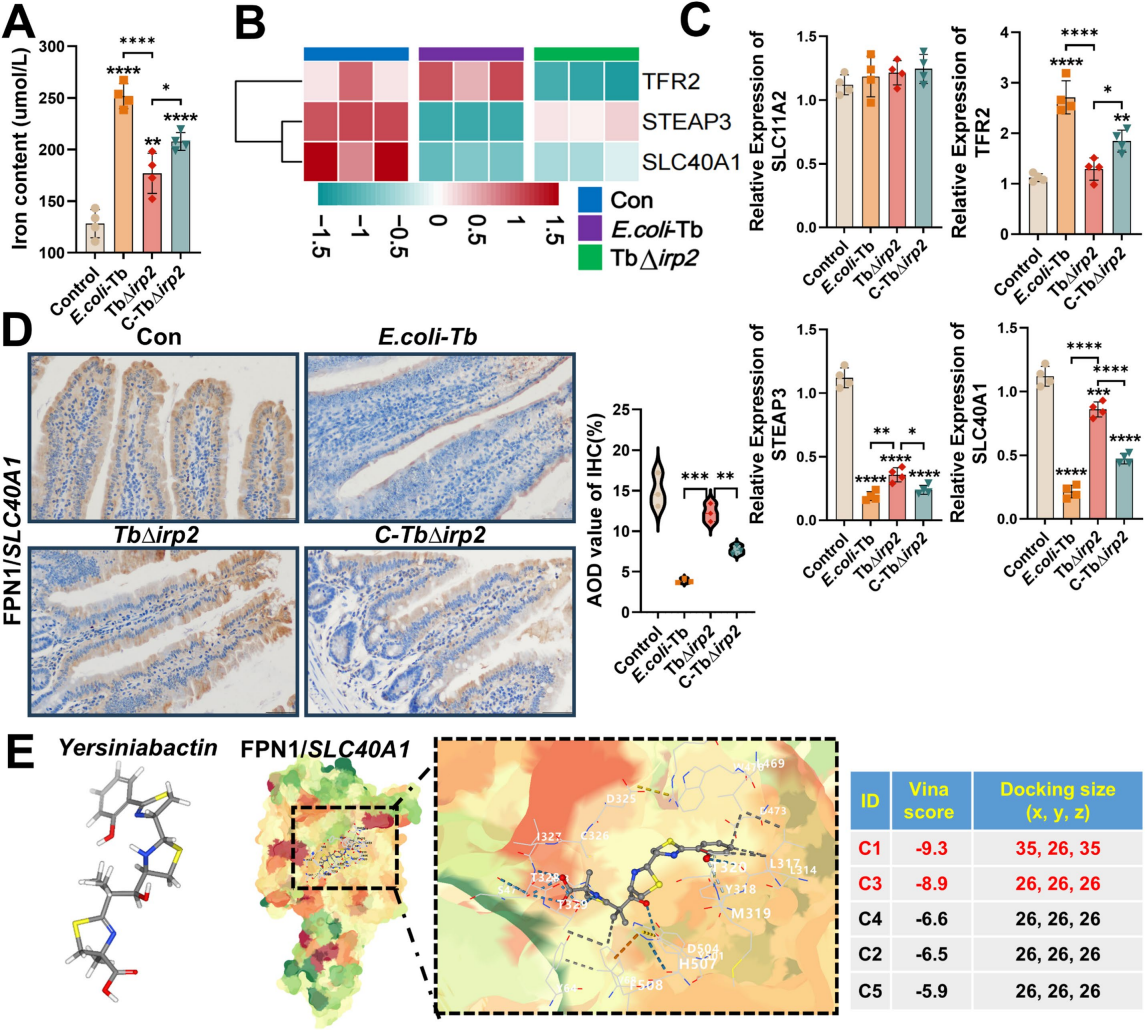
Ybt induces the accumulation of intracellular iron ions. **(A)** The observation of intracellular iron ion accumulation after various treatments. **(B)** Cluster heat maps were generated to visualize the DEGs associated with intracellular iron ion metabolism following various treatments. **(C)** qPCR was employed to ascertain the mRNA levels of *SLC11A2*, *TFR2*, *STEAP3*, and *SLC40A1*, *n* = 4. **(D)** IHC findings of various treatments (scale: 50 μm; arrows indicate typical damage) and quantification of the mean optical density of the results. **(E)** Identify the optimal binding sites of Ybt and FPN1, and to assess the docking scores obtained from different binding methodologies. ^**^p < 0.01, ^***^p < 0.001, and ^****^p < 0.0001.

The occurrence of ferroptosis is positively correlated with the intracellular accumulation of iron ions (Han et al., 2022). Consequently, we assessed the ferrous ion levels in infected cells, revealing a significant rise in *E. coli*-Tb-infected cells compared to TbΔ*irp2*-infected ones (Figure 4A). We analyzed genes associated with iron metabolism to probe the molecular mechanism underlying this Ybt-triggered iron accumulation. Interestingly, our findings revealed a decrease in the expression of FPN1 (*SLC40A1*), the primary cellular iron efflux protein (Ravasi et al., 2020; Dang et al., 2023), induced by Ybt. This downregulation was validated via Western Blot, RNA-seq and qPCR analysis (Figures 3D, 4B,C), with similar results observed in the intestinal tract of tree shrews (Figure 4D). To better understand the impact of Ybt on *SLC40A1* expression, we employed molecular docking techniques to predict the binding affinity between Ybt and FPN1. Docking simulations uncovered a robust binding interaction between Ybt and FPN1, yielding a docking score of −9.3 (Figure 4E). Collectively, these results indicate that Ybt induces ferroptosis by stimulating lipid peroxidation and intracellular iron ion accumulation. The downregulation of FPN1 expression is identified as one of the mechanisms facilitating this process.

### 3.4 Oxidized lipidomic characteristics of Ybt induced ferroptosis

**Figure 5:**
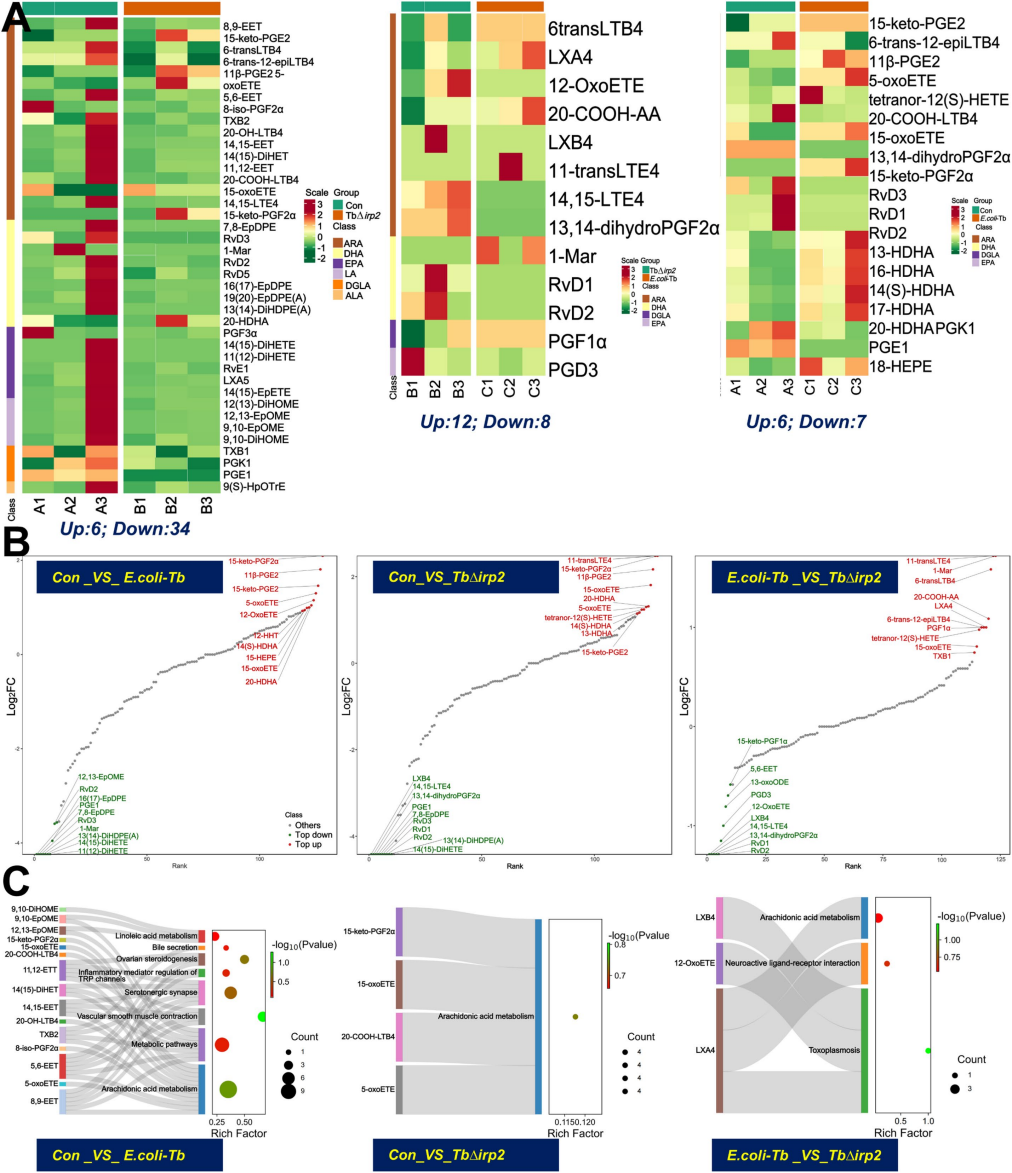
Oxidized lipidomic characteristics of Ybt induced ferroptosis. **(A)** The significant differential metabolites were selected to generate a cluster heat map to visualize the characteristics of oxidative lipid metabolites in the cells after different treatments. **(B)** The dynamic distribution map of the differences in metabolite content was drawn, with annotations provided for the top 10 down-regulated and up-regulated metabolites. **(C)** The differences of lipid oxidation metabolites in different groups were analyzed by KEGG enrichment.

Lipid peroxides are considered the main feature of ferroptosis, with PUFA being the most susceptible lipid to peroxidation during this process (Cao and Dixon, 2016). To evaluate the effect of Ybt-induced ferroptosis, we utilized LC-MS/MS to analyze oxidized lipids. Hierarchical cluster analysis was performed to assess the accumulation patterns of lipid oxide metabolites in cells following various treatments (Supplementary Figure S4A). Our results demonstrate a substantial increase in lipid peroxidation induced by Ybt, along with elevated levels of oxidative metabolites, especially those derived from ARA, docosahexaenoic acid (DHA), eicosapentaenoic acid (EPA), and dihomo-gamma-linolenic acid (DGLA). In contrast, a significant decrease in the levels of oxidative metabolites was observed after TbΔ*irp2* infection (Figure 5A). Additionally, a radar map depicting the top 1–10 metabolites with the highest fold change values was generated by comparing the quantitative results of metabolites across different groups (Supplementary Figure S5A). The findings highlight the substantial upregulation of the arachidonic acid (ARA) derivative, 6-transLTB4, by Ybt.

To enhance the understanding of metabolic differences, a dynamic distribution map was created to illustrate variations in metabolite content. The top 10 up-regulated and down-regulated metabolites were identified and labeled (Figure 5B). KEGG enrichment was subsequently conducted on the differentially expressed metabolites in each group, revealing enrichment in the ARA metabolism pathway with an up-regulation of metabolites (Figure 5C). This suggests that *E. coli* Ybt triggers oxidative stress in cells, resulting in lipid peroxidation and the subsequent generation of lipid metabolites via the ARA metabolic pathway. Additionally, correlations between various differential metabolites were assessed using Pearson correlation analysis (Supplementary Figure S4B).

A comprehensive analysis of RNA-seq and oxidative lipidomics results was undertaken to further investigate how Ybt contributes to bodily injury. Correlations between all differential genes and metabolites were calculated and visualized in a correlation cluster heat map (Supplementary Figure S5B). The analysis unveiled notable differences in the expression profiles of DEGs and metabolites after *E. coli*-Tb and TbΔ*irp2* infection. Furthermore, KEGG pathway enrichment analysis of both omics datasets indicated the collective involvement of specific DEGs and metabolites in the ARA metabolic pathway post Ybt infection (Supplementary Figure S5C). These results suggest that Ybt exacerbates lipid peroxidation in cells through the ARA pathway, ultimately promoting ferroptosis in intestinal epithelial cells.

### 3.5 6-transLTB4 promoted Ybt-induced inflammation and ferroptosis

**Figure 6:**
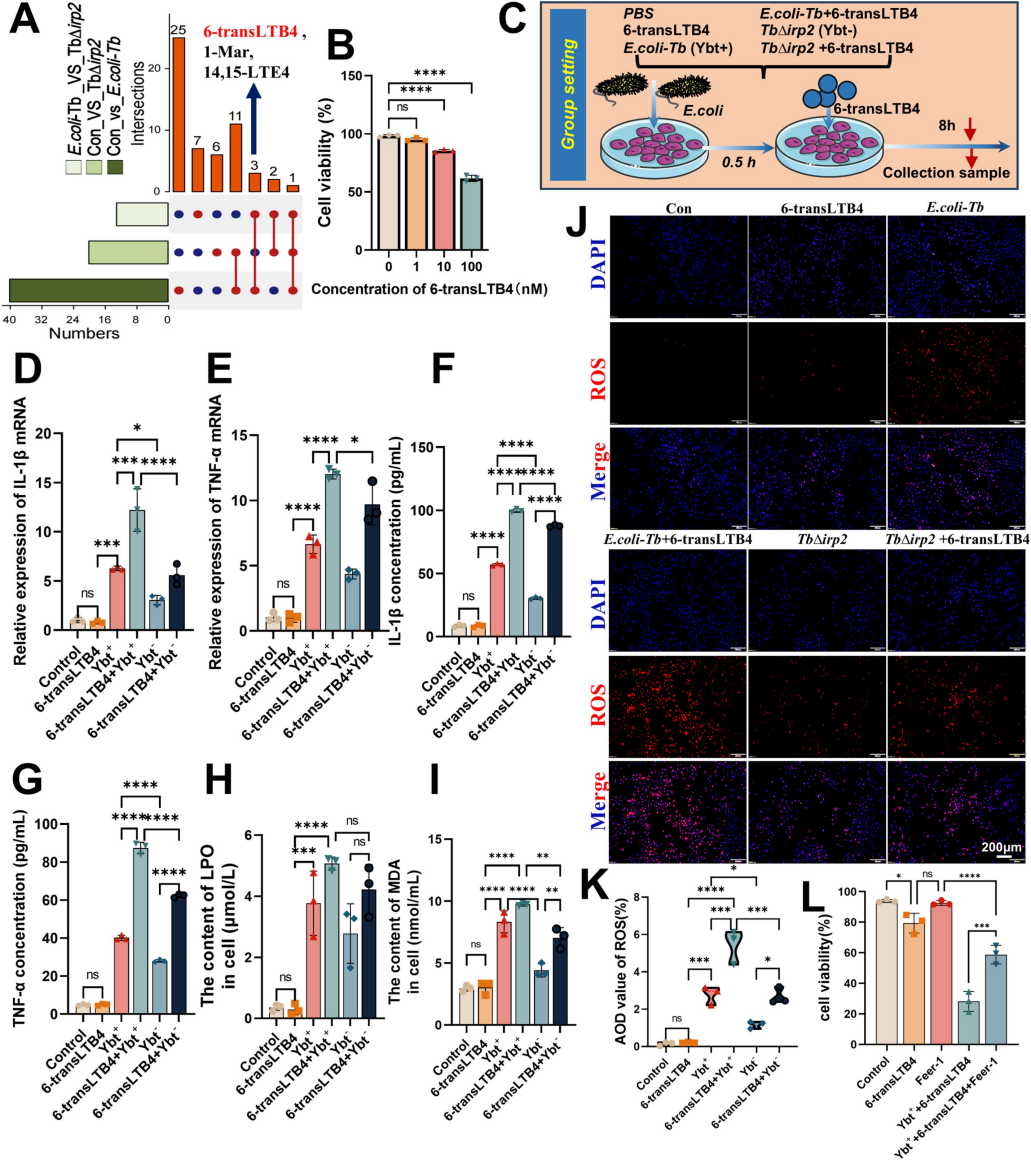
6-transLTB4 facilitated Ybt-induced inflammation and ferroptosis. **(A)** The differential metabolites after different treatments were analyzed using an Upset plot, the left side of the graph lists the names of all the collections, and the corresponding bar chart shows the total number of elements in each collection, with each row representing an independent collection and each column representing a specific crossover between the corresponding collections. **(B)** The toxicity of 6-transLTB4 to cells was assessed by measuring LDH content, *n* = 3. **(C)** Schematic representation of an exposure model involving Ybt and 6-transLTB4. **(D–G)** IL-18 and IL-1β level alterations were evaluated through both ELISA and qPCR methodologies, *n* = 3. **(H–I)** The intracellular lipid peroxide content was measured at different time points following distinct treatments, *n* = 3. **(J,K)** ROS levels in the cells were monitored using a fluorescence probe method. **(L)** The effect of the Fer-1 on cell death was observed after different treatments. ^*^p < 0.05, ^**^p < 0.01, ^***^p < 0.001, and ^****^p < 0.0001.

6-transLTB4, a pro-inflammatory lipid mediator derived from n-6 PUFA, belongs to the leukotriene family and is an isomer of LTB4. It has been shown to induce leukocyte chemotaxis and increase the production of ROS-like substances (Fretland et al., 1991; Shivaprakash et al., 2021). Interestingly, we intercrossed the differential metabolites among the Control group, *E. coli*-Tb group, and TbΔ*irp2* group and found that Ybt significantly upregulated the content of 6-transLTB4 (Figure 6A), we further explored the involvement of 6-transLTB4 in Ybt-induced inflammation and ferroptosis. We treated cells with varying doses of 6-transLTB4 and monitored cell viability over a 48-h period. After determining the non-toxic concentration of 6-transLTB4, this concentration was subsequently applied to the cells (Figures 6B,C).

**Figure 7:**
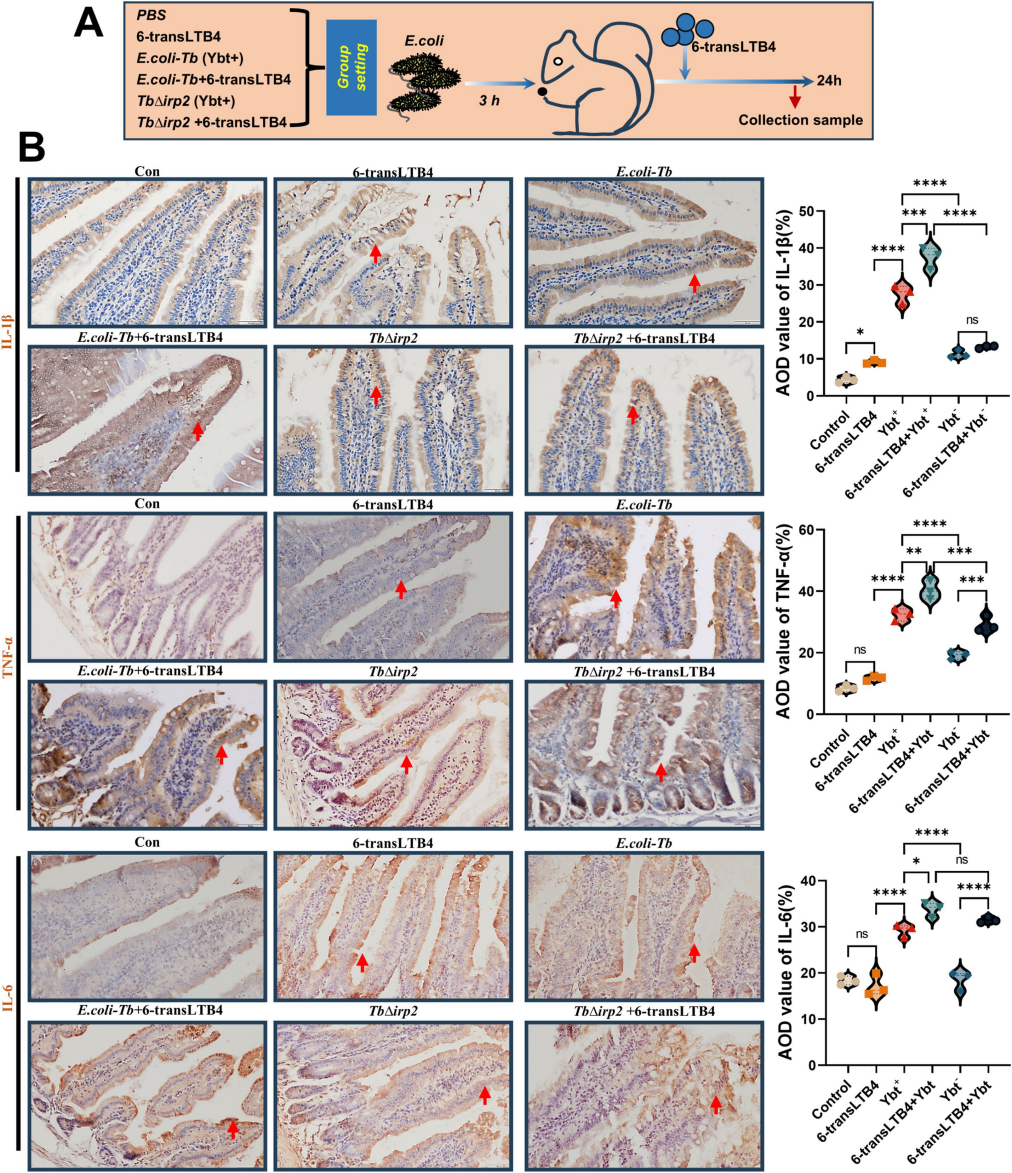
6-transLTB4 enhances Ybt-induced intestinal inflammation. **(A)** A schematic diagram of the tree shrews’ exposure model to Ybt and 6-transLTB4 is depicted. **(B)** IHC findings of various treatments (scale: 50 μm; arrows indicate typical damage) and quantification of mean optical density are presented. ^*^p < 0.05, ^**^p < 0.01, ^***^p < 0.001, and ^****^p < 0.0001.

Assays of inflammatory factors revealed that 6-transLTB4 alone had minimal impact on the inflammatory response of cells. However, co-treatment with Ybt significantly enhanced the expression and release of TNF-α and IL-1β (Figures 6D–G). Furthermore, the combination of 6-transLTB4 and Ybt induced significant lipid peroxidation damage and intracellular ROS accumulation, with 6-transLTB4 notably promoting Ybt-induced lipid peroxidation (Figures 6H–K). Notably, cell death induced by the combined treatment of 6-transLTB4 and Ybt was mitigated by the ferroptosis inhibitor Fer-1 (Figure 6L). To accomplish this, we established an *in vivo* infection model in tree shrews (Figure 7A), where the combined effects of 6-transLTB4 and *E. coli* Ybt increased the expression of inflammatory factors, leading to exacerbated intestinal inflammation in tree shrews (Figure 7B). These findings suggest that 6-transLTB4 plays a crucial role in promoting inflammation and ferroptosis induced by Ybt.

## 4 Discussion

The correlation between Ybt and *E. coli*-induced intestinal inflammation remains a topic with limited research. We observed that Ybt enhances *E. coli*-induced inflammation by upregulating the expression of inflammatory factors in the intestine. Further investigation into inflammatory mechanisms unveiled a notable enrichment of key genes in the TNF/NF-κB pathway in Ybt-stimulated intestinal epithelial cells, suggesting a close linkage between *E. coli*-induced inflammation and this pathway. The NF-κB pathway activation is governed by ROS, which subsequently triggers the expression of TNF-α and IL-1β (Siomek, 2012; Blaser et al., 2016). Moreover, ROS and cytokines establish a feedback loop through NF-κB, intensifying the inflammatory response. Bioinformatics analyses indicated a significant impact of Ybt infection on the ROS metabolic pathway, promoting ROS production. Cellular ROS equilibrium is primarily governed by antioxidative stress capacity, with the Keap1/Nrf2 pathway assuming critical importance (DeNicola et al., 2011; Hayes and Ashford, 2012). Interestingly, the study proposes that Ybt inhibits the Keap1/Nrf2 pathway, leading to intracellular ROS accumulation during Ybt infection.

Despite the well-known virulence of Ybt, its specific molecular mechanisms remain poorly understood. The Ybt iron carrier of *E. coli* is involved in activating host cell autophagy, thereby promoting the onset of Crohn’s disease (Dalmasso et al., 2021). Furthermore, Ybt promotes pyroptosis, which contributes to *E. coli*-induced intestinal inflammation via activation of the NLRP3 pathway (Wang et al., 2023b). Ferroptosis is closely linked to ROS accumulation, and its molecular mechanism differs from that of pyroptosis and autophagy (Nishizawa et al., 2021; Yang et al., 2023). Our research indicates a strong correlation between Ybt and ROS generation, leading us to propose that Ybt could trigger ferroptosis. Bioinformatics analysis provides initial support for the involvement of ferroptosis in the infection process of *E. coli*. Our study revealed that Ybt regulates the expression of key genes involved in ferroptosis, and that Ybt enhances lipid peroxidation and intracellular iron accumulation. Moreover, we observed that Fer-1 can alleviate Ybt-induced cell death, providing additional support for our hypothesis. FPN1, the main iron efflux protein in cells (Ravasi et al., 2020; Dang et al., 2023), plays a crucial role in regulating cellular iron homeostasis. Our data illustrates that Ybt suppresses FPN1 expression in cells and intestines, and has the ability to interact with FPN1. This implies that Ybt might hinder FPN1 expression through binding, leading to intracellular iron accumulation. The buildup of intracellular iron can trigger ROS production, escalate lipid peroxidation, and ultimately instigate ferroptosis. Therefore, the initiation of Ybt-induced ferroptosis may commence with the suppression of FPN1 expression. This further enhances our understanding of the mechanisms by which *E. coli* induces bodily harm.

We observed lipid oxidation metabolites induced by Ybt were predominantly present in downstream metabolites of ARA, DHA, EPA, and DGLA, with a particular emphasis on ARA metabolites. Notably, the ARA metabolic pathway is closely associated with the development of inflammation (Wang et al., 2019), suggesting that the oxidative stress induced by *E. coli* Ybt may promote inflammation by generating distinct metabolites through the ARA pathway. Furthermore, a significant upregulation of the differential metabolite 6-transLTB4 in the ARA pathway post Ybt treatment. Therefore, upon exogenous addition of 6-transLTB4, we observed a significant enhancement in Ybt-induced inflammation, ROS generation, and ferroptosis. This finding highlights the critical metabolic role of 6-transLTB4 in Ybt-mediated inflammation and ferroptosis.

Phylogenetic analysis indicates a close evolutionary relationship between tree shrews and humans, particularly with primates (Fan et al., 2013). Moreover, high-throughput analyses reveal a significant similarity between the proteomic compositions of muscle and liver tissues in tree shrews and humans (Li et al., 2012). Therefore, studying the effects of *E. coli* on tree shrew intestinal health can provide valuable evidence for understanding *E. coli*-induced intestinal damage in humans.

## 5 Conclusion

**Figure 8:**
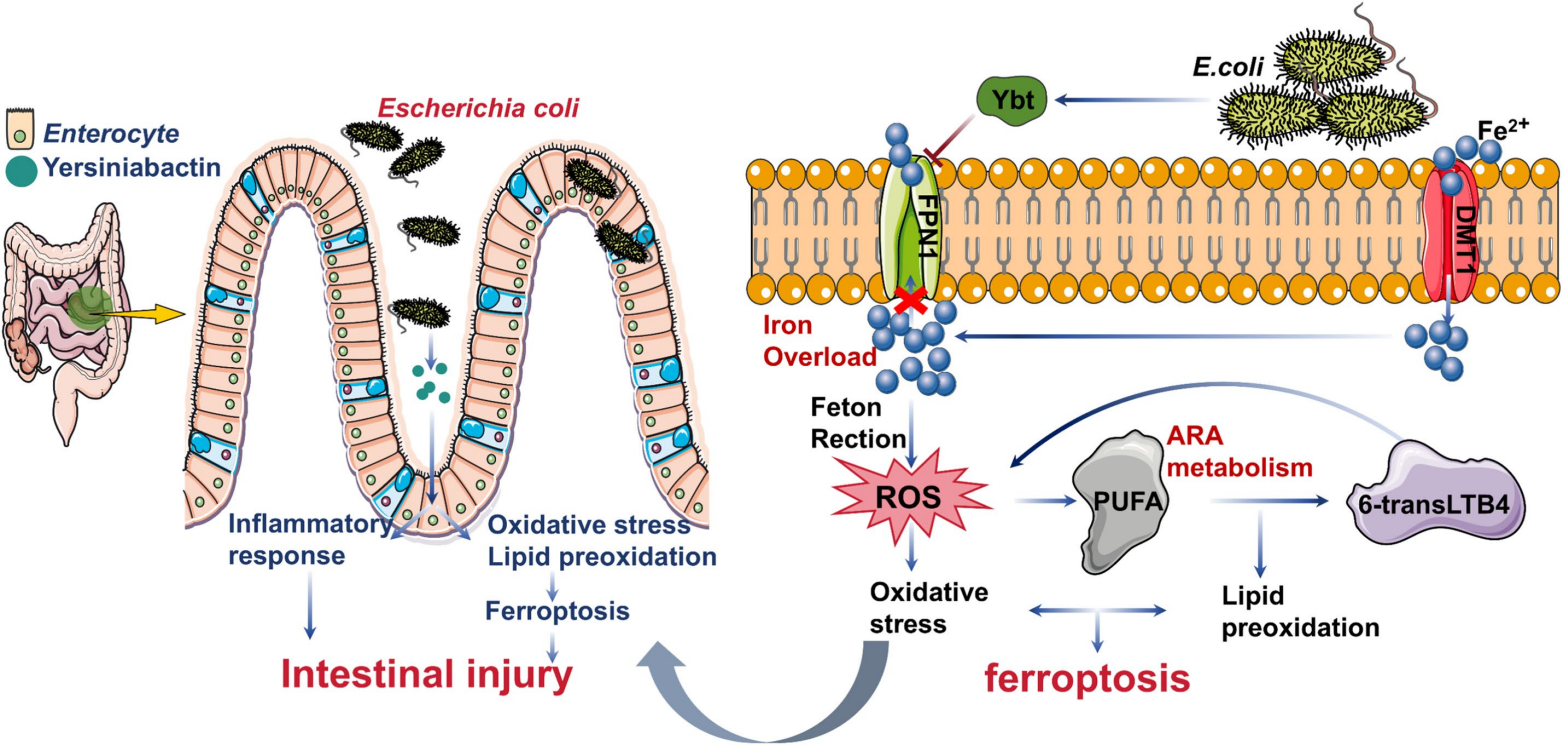
Schematic representation of the molecular mechanism of the article.

In conclusion, ferroptosis induced by *E. coli*-Ybt exacerbates *E. coli*-induced intestinal inflammation by promoting ROS production, activating the TNF/NF-κB pathway, and suppressing FPN1 expression. Our findings also reveal that oxidative metabolites generated by Ybt via the ARA metabolic pathway, particularly 6-transLTB4, significantly enhance inflammation and ferroptosis (Figure 8). However, a limitation of this study is the incomplete understanding of how Ybt specifically modulates the interaction between ROS and iron homeostasis. Future research should focus on elucidating the detailed molecular mechanisms underlying these processes. Overall, Ybt plays a pivotal role in *E. coli*-induced intestinal injury, offering valuable insights into its novel pathogenic mechanisms.

## Data Availability

Data for high-throughput sequencing generated in this study have been deposited at BioProject (PRJNA1097322).

## Footnotes

^1^ http://www.omicshare.com/tools

^2^ http://www.metware.cn/

^3^ https://cloud.metware.cn

## References

[ref1] Bachman M. Oyler J. Burns S. Caza M. Lépine F. Dozois C.. (2011). *Klebsiella pneumoniae* yersiniabactin promotes respiratory tract infection through evasion of lipocalin 2. Infect. Immun. 79, 3309–3316. doi: 10.1128/iai.05114-11, PMID: 21576334 PMC3147564

[ref2] Bersuker K. Hendricks J. Li Z. Magtanong L. Ford B. Tang P.. (2019). The CoQ oxidoreductase FSP1 acts parallel to GPX4 to inhibit ferroptosis. Nature 575, 688–692. doi: 10.1038/s41586-019-1705-2, PMID: 31634900 PMC6883167

[ref3] Blaser H. Dostert C. Mak T. Brenner D. (2016). TNF and ROS crosstalk in inflammation. Trends Cell Biol. 26, 249–261. doi: 10.1016/j.tcb.2015.12.002 26791157

[ref4] Cao J. Dixon S. (2016). Mechanisms of ferroptosis. Cell. Mol. Life. Sci. 73, 2195–2209. doi: 10.1007/s00018-016-2194-1, PMID: 27048822 PMC4887533

[ref5] Carniel E. Guilvout I. Prentice M. (1996). Characterization of a large chromosomal “high-pathogenicity island” in biotype 1B *Yersinia enterocolitica*. J. Bacteriol. 178, 6743–6751. doi: 10.1128/jb.178.23.6743-6751.1996, PMID: 8955291 PMC178570

[ref6] Dalmasso G. Nguyen H. Faïs T. Massier S. Chevarin C. Vazeille E.. (2021). Yersiniabactin siderophore of Crohn’s disease-associated adherent-invasive *Escherichia coli* is involved in autophagy activation in host cells. Int. J. Mol. Sci. 22:3512. doi: 10.3390/ijms22073512, PMID: 33805299 PMC8037853

[ref7] Dang Q. Huang C. Liang Y. Hewawasam S. Liu L. (2023). SLC40A1 is involved in iron accumulation in human lung fibroblasts. Physiology 38:5729570. doi: 10.1152/physiol.2023.38.S1.5729570

[ref8] DeNicola G. Karreth F. Humpton T. Gopinathan A. Wei C. Frese K.. (2011). Oncogene-induced Nrf2 transcription promotes ROS detoxification and tumorigenesis. Nature 475, 106–109. doi: 10.1038/nature10189, PMID: 21734707 PMC3404470

[ref9] Dixon S. Lemberg K. Lamprecht M. Skouta R. Zaitsev E. Gleason C.. (2012). Ferroptosis: an iron-dependent form of nonapoptotic cell death. Cell 149, 1060–1072. doi: 10.1016/j.cell.2012.03.042, PMID: 22632970 PMC3367386

[ref10] Doll S. Freitas F. Shah R. Aldrovandi M. da M. Ingold I.. (2019). FSP1 is a glutathione-independent ferroptosis suppressor. Nature 575, 693–698. doi: 10.1038/s41586-019-1707-0, PMID: 31634899

[ref11] Ellermann M. Gharaibeh R. Fulbright L. Dogan B. Moore L. Broberg C.. (2019). Yersiniabactin-producing adherent/invasive *Escherichia coli* promotes inflammation-associated fibrosis in gnotobiotic mice. Infect. Immun. 87:e00587. doi: 10.1128/iai.00587-19, PMID: 31481410 PMC6803345

[ref12] Fan Y. Huang Z. Y. Cao C. C. Chen C. S. Chen Y. X. Fan D. D.. (2013). Genome of the Chinese tree shrew. Nat. Commun. 4:1426. doi: 10.1038/ncomms2416, PMID: 23385571

[ref13] Fock E. Bachteeva V. Lavrova E. Parnova R. (2018). Mitochondrial-targeted antioxidant MitoQ prevents *E. coli* lipopolysaccharide-induced accumulation of triacylglycerol and lipid droplets biogenesis in epithelial cells. J. Lipids 2018:5745790. doi: 10.1155/2018/5745790, PMID: 30245885 PMC6139225

[ref14] Fretland D. Widomski D. Anglin C. Walsh R. Levin S. Gaginella T. (1991). 6-trans-leukotriene B4 is a neutrophil chemotaxin in the guinea pig dermis. J. Leukoc. Biol. 49, 283–288. doi: 10.1002/jlb.49.3.283 1847716

[ref15] Garcia E. Brumbaugh A. Mobley H. (2011). Redundancy and specificity of *Escherichia coli* iron acquisition systems during urinary tract infection. Infect. Immun. 79, 1225–1235. doi: 10.1128/iai.01222-10, PMID: 21220482 PMC3067483

[ref16] Govindarajan D. K. Kandaswamy K. (2022). Virulence factors of uropathogens and their role in host pathogen interactions. Cell Surf. 8:100075. doi: 10.1016/j.tcsw.2022.100075, PMID: 35198842 PMC8841375

[ref17] Govindarajan D. K. Viswalingam N. Meganathan Y. Kandaswamy K. (2020). Adherence patterns of *Escherichia coli* in the intestine and its role in pathogenesis. Med. Microecol. 5:100025. doi: 10.1016/j.medmic.2020.100025

[ref18] Han Y. Dong Z. Wang C. Li Q. Hao Y. Yang Z.. (2022). Ferrous ions doped calcium carbonate nanoparticles potentiate chemotherapy by inducing ferroptosis. J. Control. Release 348, 346–356. doi: 10.1016/j.jconrel.2022.06.002, PMID: 35679965

[ref19] Hayes J. Ashford M. (2012). Nrf2 orchestrates fuel partitioning for cell proliferation. Cell Metab. 16, 139–141. doi: 10.1016/j.cmet.2012.07.009, PMID: 22883227

[ref20] Hsu H. Xiong J. Goeddel D. (1995). The TNF receptor 1-associated protein TRADD signals cell death and NF-kappa B activation. Cell 81, 495–504. doi: 10.1016/0092-8674(95)90070-5, PMID: 7758105

[ref21] Ingold I. Berndt C. Schmitt S. Doll S. Poschmann G. Buday K.. (2018). Selenium utilization by GPX4 is required to prevent hydroperoxide-induced ferroptosis. Cell 172, 409–422.e21. doi: 10.1016/j.cell.2017.11.048, PMID: 29290465

[ref22] Jiang Y. Chen B. Duan C. Sun B. Yang J. Yang S. (2015). Multigene editing in the *Escherichia coli* genome via the CRISPR-Cas9 system. Appl. Environ. Microbiol. 81, 2506–2514. doi: 10.1128/aem.04023-14 25636838 PMC4357945

[ref23] Jiang X. Stockwell B. Conrad M. (2021). Ferroptosis: mechanisms, biology and role in disease. Nat. Rev. Mol. Cell Biol. 22, 266–282. doi: 10.1038/s41580-020-00324-8, PMID: 33495651 PMC8142022

[ref24] Koh E. Robinson A. Bandara N. Rogers B. Henderson J. (2017). Copper import in *Escherichia coli* by the yersiniabactin metallophore system. Nat. Chem. Biol. 13, 1016–1021. doi: 10.1038/nchembio.2441, PMID: 28759019 PMC5562518

[ref25] Li R. Xu W. Wang Z. Liang B. Wu J. R. Zeng R. (2012). Proteomic characteristics of the liver and skeletal muscle in the Chinese tree shrew (*Tupaia belangeri chinensis*). Protein Cell 3, 691–700. doi: 10.1007/s13238-012-2039-0, PMID: 22886497 PMC4875369

[ref26] Liu C. Shan C. Dong Q. Fu G. Zhao R. Yan Y.. (2018). Pathogenic *E. coli* HPI upregulate the expression of inflammatory factors in porcine small intestinal epithelial cells by ubiquitin proteasome pathway. Res. Vet. Sci. 120, 41–46. doi: 10.1016/j.rvsc.2018.08.009, PMID: 30199780

[ref27] Liu Y. Yang X. Gan J. Chen S. Xiao Z.-X. Cao Y. (2022). CB-Dock2: improved protein–ligand blind docking by integrating cavity detection, docking and homologous template fitting. Nucleic Acids Res. 50, W159–W164. doi: 10.1093/nar/gkac394, PMID: 35609983 PMC9252749

[ref28] Magistro G. Magistro C. Stief C. G. Schubert S. (2017). The high-pathogenicity island (HPI) promotes flagellum-mediated motility in extraintestinal pathogenic *Escherichia coli*. PLoS One 12:e0183950. doi: 10.1371/journal.pone.0183950, PMID: 29016611 PMC5634559

[ref29] Morris G. Gevezova M. Sarafian V. Maes M. (2022). Redox regulation of the immune response. Cell. Mol. Immunol. 19, 1079–1101. doi: 10.1038/s41423-022-00902-0, PMID: 36056148 PMC9508259

[ref30] Nishizawa H. Matsumoto M. Chen G. Ishii Y. Tada K. Onodera M.. (2021). Lipid peroxidation and the subsequent cell death transmitting from ferroptotic cells to neighboring cells. Cell Death Dis. 12:332. doi: 10.1038/s41419-021-03613-y, PMID: 33782392 PMC8007748

[ref31] Pham C. Bubici C. Zazzeroni F. Papa S. Jones J. Alvarez K.. (2004). Ferritin heavy chain upregulation by NF-kappaB inhibits TNFalpha-induced apoptosis by suppressing reactive oxygen species. Cell 119, 529–542. doi: 10.1016/j.cell.2004.10.017, PMID: 15537542

[ref32] Portolés M. Ainaga M. Pagani R. (1993). The induction of lipid peroxidation by *E. coli* lipopolysaccharide on rat hepatocytes as an important factor in the etiology of endotoxic liver damage. Biochim. Biophys. Acta 1158, 287–292. doi: 10.1016/0304-4165(93)90027-6, PMID: 8251529

[ref33] Ravasi G. Pelucchi S. Russo A. Mariani R. Piperno A. (2020). Ferroportin disease: a novel SLC40A1 mutation. Dig. Liver Dis. 52, 688–690. doi: 10.1016/j.dld.2020.03.013, PMID: 32360131

[ref34] Rochette L. Dogon G. Rigal E. Zeller M. Cottin Y. Vergely C. (2022). Lipid peroxidation and Iron metabolism: two corner stones in the homeostasis control of ferroptosis. Int. J. Mol. Sci. 24:449. doi: 10.3390/ijms24010449, PMID: 36613888 PMC9820499

[ref35] Shivaprakash K. Sen S. Paul S. Kiesecker J. Bawa K. (2021). Mammals, wildlife trade, and the next global pandemic. Curr. Biol. 31, 3671–3677.e3. doi: 10.1016/j.cub.2021.06.006, PMID: 34237267

[ref36] Siomek A. (2012). NF-κB signaling pathway and free radical impact. Acta Biochim. Pol. 59, 323–331. doi: 10.18388/abp.2012_2116, PMID: 22855720

[ref37] Sobieszczańska B. (2008). Distribution of genes encoding iron uptake systems among enteroaggregative *Escherichia coli* strains isolated from adults with irritable bowel syndrome. Clin. Microbiol. Infect. 14, 1083–1086. doi: 10.1111/j.1469-0691.2008.02093.x, PMID: 19040481

[ref38] Stockwell B. (2022). Ferroptosis turns 10: emerging mechanisms, physiological functions, and therapeutic applications. Cell 185, 2401–2421. doi: 10.1016/j.cell.2022.06.003, PMID: 35803244 PMC9273022

[ref39] Stockwell B. Friedmann J. Bayir H. Bush A. Conrad M. Dixon S.. (2017). Ferroptosis: a regulated cell death Nexus linking metabolism, redox biology, and disease. Cell 171, 273–285. doi: 10.1016/j.cell.2017.09.021, PMID: 28985560 PMC5685180

[ref40] Tang W. Wang W. Zhang Y. Liu S. Liu Y. Zheng D. (2009). TRAIL receptor mediates inflammatory cytokine release in an NF-kappaB-dependent manner. Cell Res. 19, 758–767. doi: 10.1038/cr.2009.57, PMID: 19434100

[ref41] Wang T. Fu X. Chen Q. Patra J. K. Wang D. Wang Z.. (2019). Arachidonic acid metabolism and kidney inflammation. Int. J. Mol. Sci. 20:3683. doi: 10.3390/ijms20153683, PMID: 31357612 PMC6695795

[ref42] Wang H. Lv L.-B. Chen L.-P. Xiao J.-L. Shen J. Gao B.. (2023a). Hemolysin co-regulatory protein 1 enhances the virulence of clinically isolated *Escherichia coli* in KM mice by increasing inflammation and inducing pyroptosis. Toxins 15:171. doi: 10.3390/toxins15030171, PMID: 36977062 PMC10058142

[ref43] Wang H. Shan C.-L. Gao B. Xiao J.-L. Shen J. Zhao J.-G.. (2023b). Yersiniabactin-producing *E. coli* induces the pyroptosis of intestinal epithelial cells via the NLRP3 pathway and promotes gut inflammation. Int. J. Mol. Sci. 24:11451. doi: 10.3390/ijms241411451, PMID: 37511208 PMC10380849

[ref44] Wang F. Sun H. Kang C. Yan J. Chen J. Feng X.. (2024). Genomic island-encoded regulatory proteins in enterohemorrhagic *Escherichia coli* O157:H7. Virulence 15:2313407. doi: 10.1080/21505594.2024.2313407 38357901 PMC10877973

[ref45] Yang X. Chen Y. Guo J. Li J. Zhang P. Yang H.. (2023). Polydopamine nanoparticles targeting Ferroptosis mitigate intervertebral disc degeneration via reactive oxygen species depletion, iron ions chelation, and GPX4 ubiquitination suppression. Adv. Sci. 10:e2207216. doi: 10.1002/advs.202207216, PMID: 36951540 PMC10161035

[ref46] Zhang Y. Yang Y. Chen W. Mi C. Xu X. Shen Y.. (2023). BaP/BPDE suppressed endothelial cell angiogenesis to induce miscarriage by promoting MARCHF1/GPX4-mediated ferroptosis. Environ. Int. 180:108237. doi: 10.1016/j.envint.2023.108237 37802009

