# Supplementary material for: Yersiniabactin produced by Escherichia coli promotes intestinal inflammation through lipid peroxidation and ferroptosis
Source: Front Microbiol. 2025 Feb 17;16:1542801. doi: 10.3389/fmicb.2025.1542801 (PMC11872927; doi:10.3389/fmicb.2025.1542801)
Supplement: Supplementary file 1 [file Data_Sheet_1.docx]

Supplementary Material

## Supplementary Tables

Table S1 Primers for qPCR analysis to detect mRNA levels

| Gene symbol | Sequence (5' - 3') |
| --- | --- |
| *TNF-α*  NM_000594 | CCTCTCTCTAATCAGCCCTCTG |
|  | GAGGACCTGGGAGTAGATGAG |
| *IL-1β*  NM_000576 | ATGATGGCTTATTACAGTGGCAA |
|  | GTCGGAGATTCGTAGCTGGA |
| *PTGS2*  NM_000963 | CTGGCGCTCAGCCATACAG |
|  | CGCACTTATACTGGTCAAATCCC |
| *HMOX1*  NM_002133 | AAGACTGCGTTCCTGCTCAAC |
|  | AAAGCCCTACAGCAACTGTCG |
| *GAPDH*  NM_002046 | CAGGAGGCATTGCTGATGAT |
|  | GAAGGCTGGGGCTCATTT |
| *ACSL4*  NM_004458 | CATCCCTGGAGCAGATACTCT |
|  | TCACTTAGGATTTCCCTGGTCC |
| *ACSL3*  NM_004457 | GCCGAGTGGATGATAGCTGC |
|  | ATGGCTGGACCTCCTAGAGTG |
| *FASN*  NM_004104 | AAGGACCTGTCTAGGTTTGATGC |
|  | TGGCTTCATAGGTGACTTCCA |
| *GPX4*  NM_001039847 | GAGGCAAGACCGAAGTAAACTAC |
|  | CCGAACTGGTTACACGGGAA |
| *GCH1*  NM_001024024 | ACGAGCTGAACCTCCCTAAC |
|  | GAACCAAGTGATGCTCACACA |
| *CYBB*  NM_000397 | ACCGGGTTTATGATATTCCACCT |
|  | GATTTCGACAGACTGGCAAGA |

## Supplementary Figures


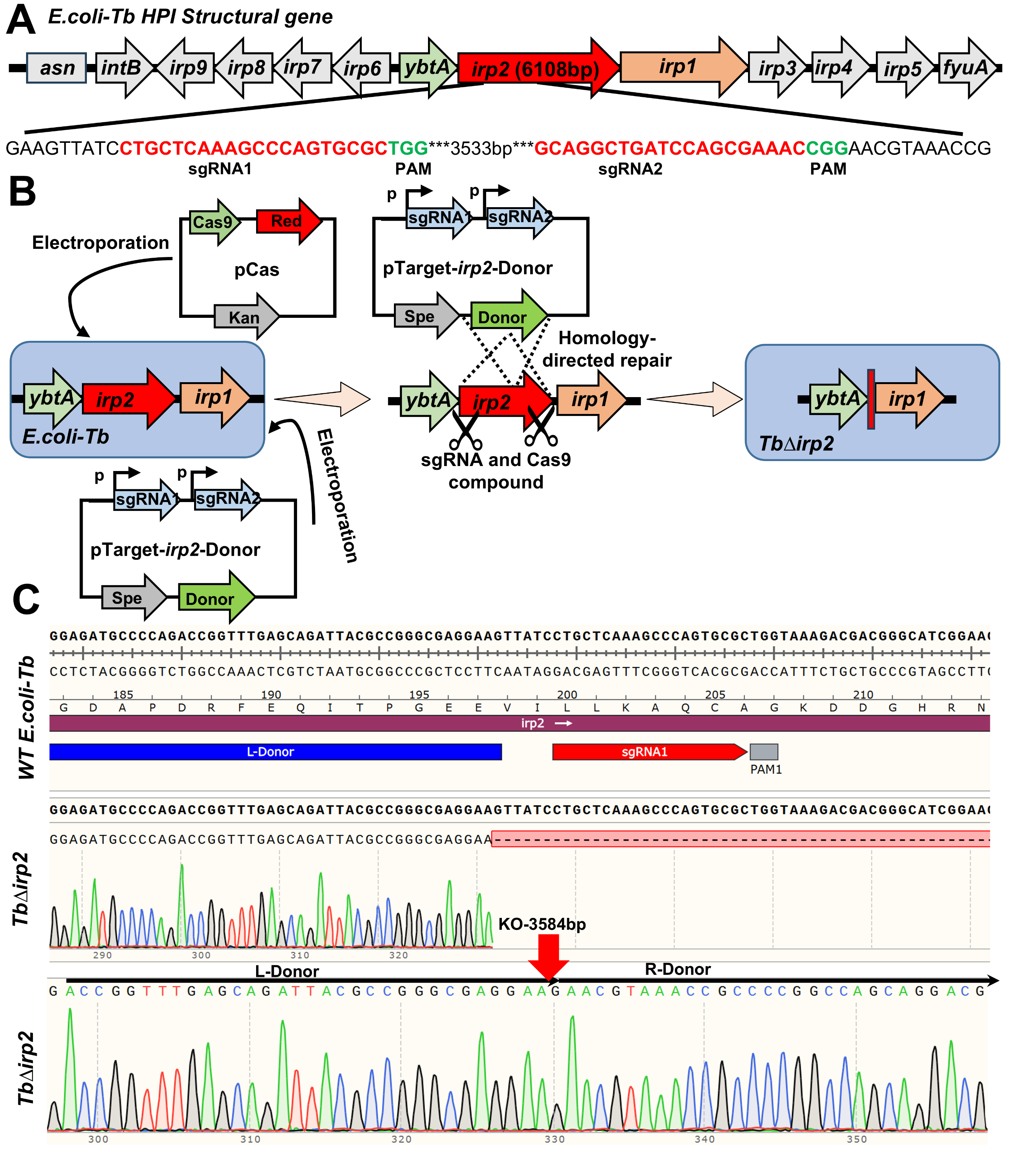


**Supplementary Figure 1. Construction and identification of *irp2* gene knockout strain.** (**A)** The gene structure of the High Pathogenicity Island (HPI) and the checkpoint of *irp2*-sgRNA. The 3533bp base that was left out is denoted by the symbol ***. (**B)** A schematic diagram depicting the *irp2* gene knockout, with the homologous recombination of gene segments in the *irp2* gene knockout strategy illustrated by the dashed line. (**C)** Sequencing results of the *irp2* gene knockout.


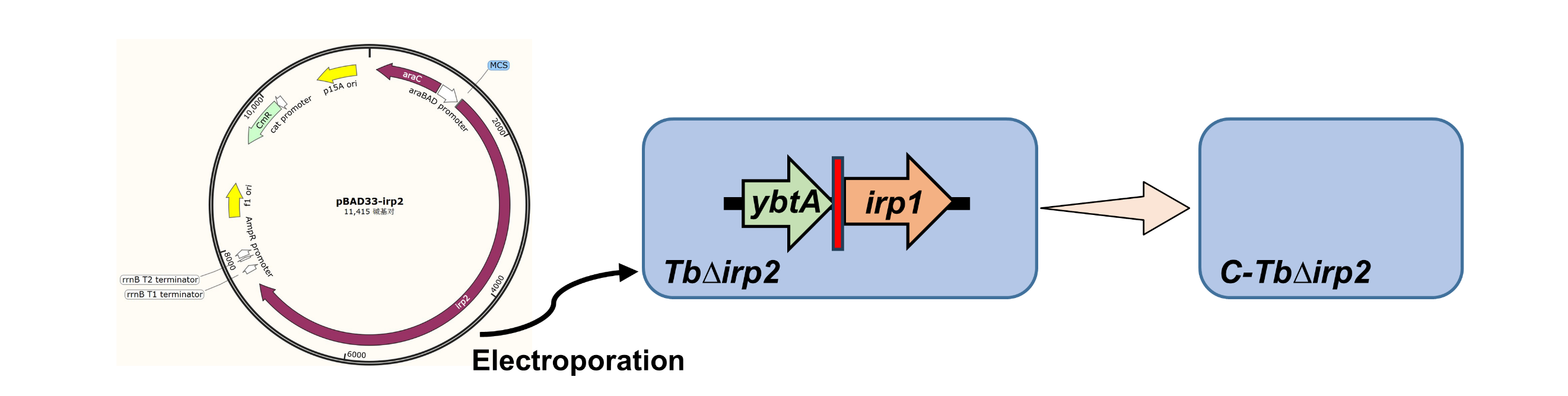


**Supplementary Figure 2 Construction of TbΔ*irp2* mutant complement strains.**

**
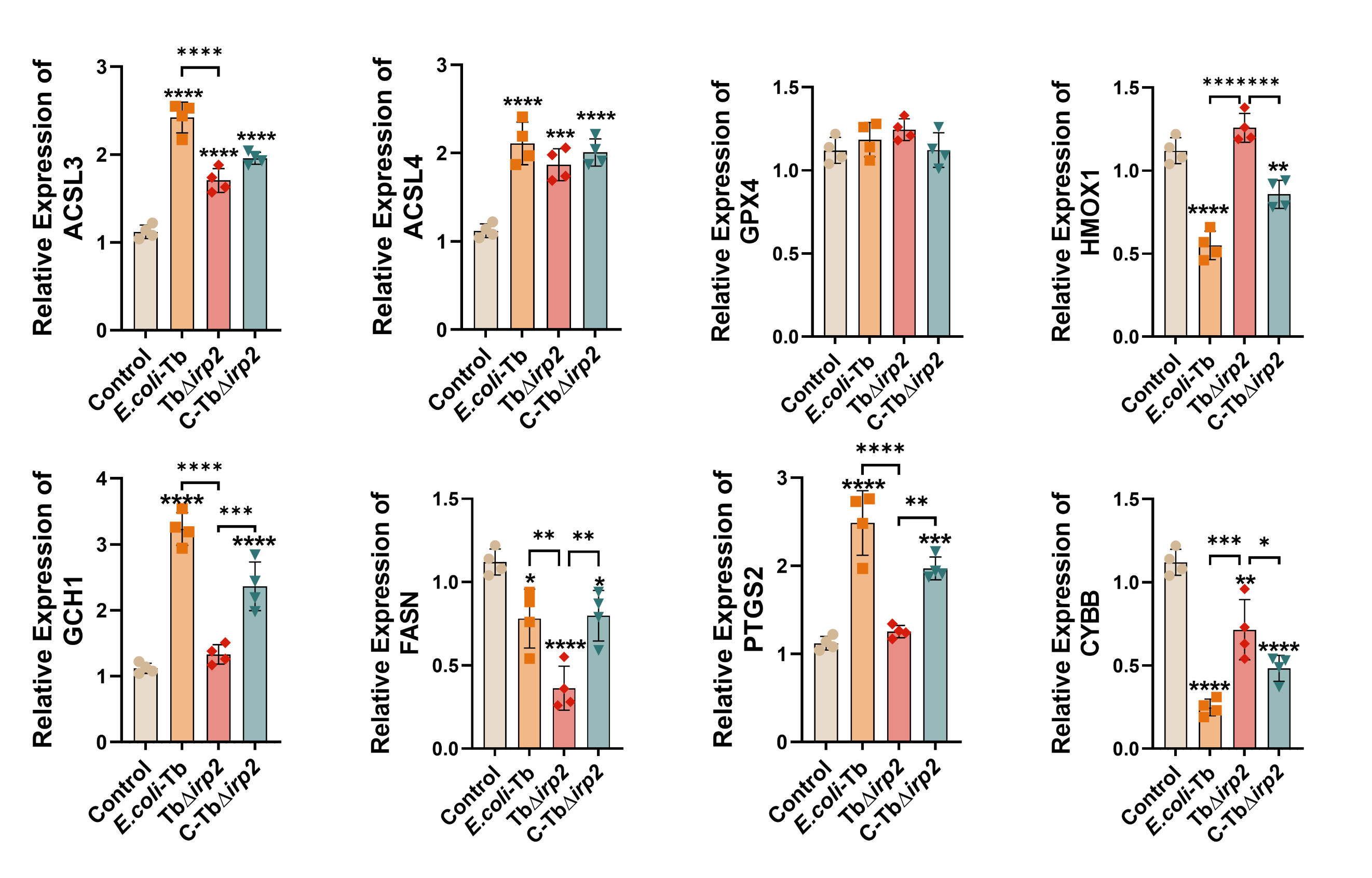
**

**Supplementary Figure 3. qPCR detection of ferroptosis marker genes after *E. coli* infection.** * *p* < 0.05, ** *p* < 0.01, *** *p* < 0.001, and **** *p* < 0.0001.


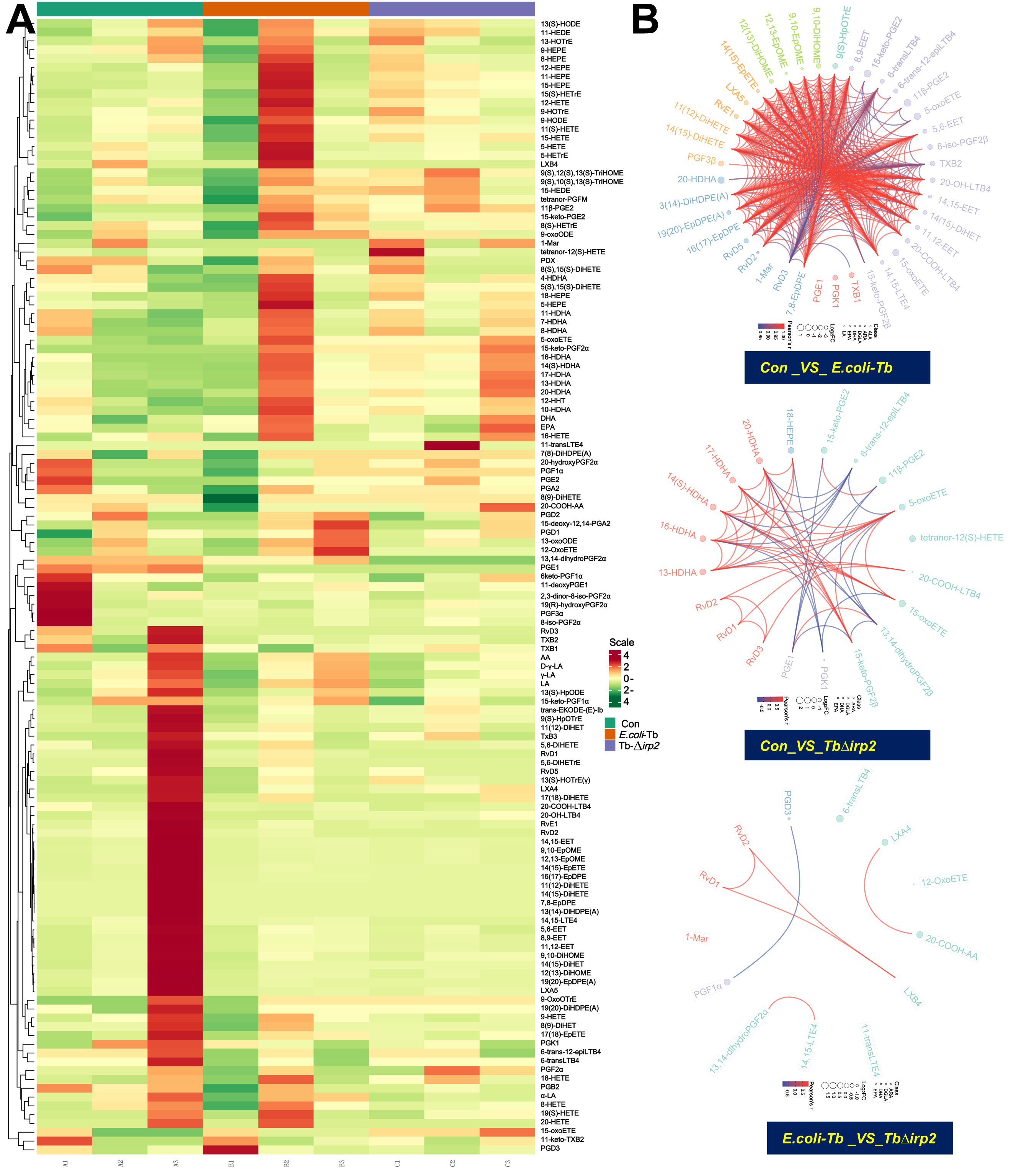


**Supplementary Figure 4. Targeted oxidative lipidomic analysis of cells after different treatments.** (**A)** Cluster heatmap analysis of differential metabolites in all samples (red represents high content, green represents low content). (**B)** Pearson correlation analysis was used to analyze the correlation of metabolites with significant differences screened according to the screening criteria. The size of the dot in the figure represents the size of the Log2FC value, with larger dots indicating larger Log2FC values. The color of the dots represents the source classification of differential metabolites in this group of comparisons, and the line represents the correlation value of metabolites at the corresponding position.


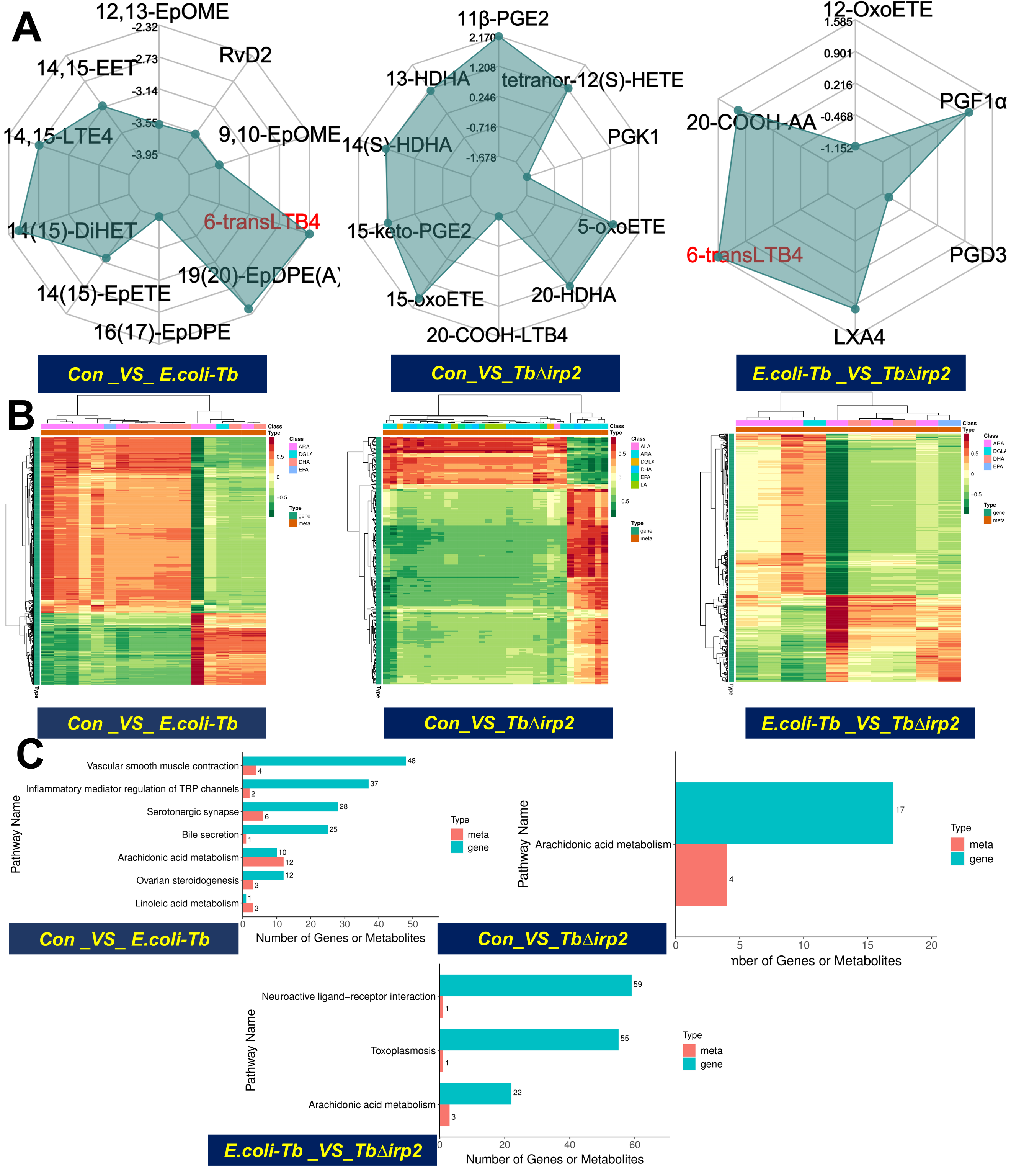


**Supplementary Figure 5. Combined analysis of RNA-seq and targeted oxidized lipidomics.** (**A)** The differences in quantitative results of metabolites among different groups were calculated. The top 10 metabolites with the largest fold change value were selected to draw the radar map. The grid lines on the radar map correspond to the fold change values, and the green shadow is composed of the lines representing the fold change values for each substance. (**B)** Correlation results of differential genes and differential metabolites were selected, and a correlation cluster heatmap was generated. Each row represents a gene, and each column represents a metabolite. Red indicates a positive correlation between genes and metabolites, while green indicates a negative correlation. (**C)** KEGG pathways enriched by the two omics were plotted as a bar graph, depicting the number of differential metabolites and differential genes enriched in each pathway. The y-axis represents the KEGG pathway name, with red and green bars representing the metabolome and transcriptome, respectively.
